# Scientific principles for the identification of endocrine-disrupting chemicals: a consensus statement

**DOI:** 10.1007/s00204-016-1866-9

**Published:** 2016-10-06

**Authors:** Roland Solecki, Andreas Kortenkamp, Åke Bergman, Ibrahim Chahoud, Gisela H. Degen, Daniel Dietrich, Helmut Greim, Helen Håkansson, Ulla Hass, Trine Husoy, Miriam Jacobs, Susan Jobling, Alberto Mantovani, Philip Marx-Stoelting, Aldert Piersma, Vera Ritz, Remy Slama, Ralf Stahlmann, Martin van den Berg, R. Thomas Zoeller, Alan R. Boobis

**Affiliations:** 1 Federal Institute for Risk Assessment, Berlin, Germany; 2 Institute of Environment, Health and Societies, Brunel University, London, Uxbridge, UK; 3 Swedish Toxicology Sciences Research Center, Södertälje, Sweden; 4 Charité, Berlin, Germany; 5 IFADO, Dortmund, Germany; 6 Universität Konstanz, Konstanz, Germany; 7 TU München, München, Germany; 8 Institute of Environmental Medicine, Karolinska Institutet, Stockholm, Sweden; 9 Technical University of Denmark, DTU, Søborg, Denmark; 10 Norwegian Institute of Public Health, Oslo, Norway; 11 Public Health England, Chilton, UK; 12 Istituto Superiore di Sanità, Rome, Italy; 13 RIVM, Bilthoven, The Netherlands; 14 Inserm, CNRS and University Grenoble-Alpes Joint Research Centre, Grenoble, France; 15 Institute of Risk Assessment Studies (IRAS), Utrecht University, Utrecht, The Netherlands; 16 University of Massachusetts, Amherst, MA USA; 17 Imperial College London, London, UK

## Abstract

Endocrine disruption is a specific form of toxicity, where natural and/or anthropogenic chemicals, known as “endocrine disruptors” (EDs), trigger adverse health effects by disrupting the endogenous hormone system. There is need to harmonize guidance on the regulation of EDs, but this has been hampered by what appeared as a lack of consensus among scientists. This publication provides summary information about a consensus reached by a group of world-leading scientists that can serve as the basis for the development of ED criteria in relevant EU legislation. Twenty-three international scientists from different disciplines discussed principles and open questions on ED identification as outlined in a draft consensus paper at an expert meeting hosted by the German Federal Institute for Risk Assessment (BfR) in Berlin, Germany on 11–12 April 2016. Participants reached a consensus regarding scientific principles for the identification of EDs. The paper discusses the consensus reached on background, definition of an ED and related concepts, sources of uncertainty, scientific principles important for ED identification, and research needs. It highlights the difficulty in retrospectively reconstructing ED exposure, insufficient range of validated test systems for EDs, and some issues impacting on the evaluation of the risk from EDs, such as non-monotonic dose–response and thresholds, modes of action, and exposure assessment. This report provides the consensus statement on EDs agreed among all participating scientists. The meeting facilitated a productive debate and reduced a number of differences in views. It is expected that the consensus reached will serve as an important basis for the development of regulatory ED criteria.

## Introduction

In summer 2013, when an initial draft of criteria for the identification of endocrine disrupters (EDs) was discussed within the European Commission (the executive of the European Union), a group of toxicology journal editors published severe scientific concerns about the proposed approaches (Dietrich et al. 2013). As a result, a controversy about the toxicological principles that should guide the identification of endocrine-disrupting chemicals flared up in the scientific press among toxicologists and endocrinologists (Bergman et al. 2013; Zoeller et al. 2014; Autrup et al. 2015). The realization that this dispute had contributed to a degree of misunderstanding among decision-makers in the European Commission motivated a group of those involved in the debates to explore whether it might be possible to bridge the differing views and come to a common understanding. In this brief communication, we describe the political and regulatory context that has led to this debate, and present the consensus that has been reached among scientists who took opposing views in the previous dispute, during a two-day workshop held in Berlin, Germany, 11–12 April 2016, hosted by the German Federal Institute for Risk Assessment (BfR).

In line with established practice in other jurisdictions, the risk management of chemicals in the EU is generally based on risk characterization. However, for some toxicological effects, the EU has introduced hazard-based regulations. This applies especially to chemicals used as active substances in plant protection products and biocidal products. According to provisions in several pieces of EU law for plant protection products and biocidal products, the European Commission was obliged to develop scientific criteria for the identification of EDs by 2013, but to date (April 2016), has failed to do so.

Motivated by concerns about health effects caused by the delay in developing criteria for endocrine-disrupting substances, Sweden (a member state of the EU) brought a court case against the European Commission in 2014. Finally, in winter 2015, the General Court of the EU ruled that the Commission had breached EU law, by failing to adopt measures concerning the specification of scientific criteria for the identification of endocrine-disrupting substances. The Court further noted that the Commission’s defense that the scientific criteria which it had proposed were the subject of criticism, in summer 2013, was irrelevant to the fact that the Commission had an obligation to present these criteria according to the deadlines enshrined in EU law (December 2013) (General Court of the EU 2015).

The apparent controversy among scientists centered on disagreements about the most appropriate approach to assess endocrine disruptors, and was focused on the scientific assumptions that could be made during the identification of a chemical as an endocrine disruptor. Prominent in these disputes was the question of the existence of thresholds for endocrine disruptors and of the significance of non-monotonic dose–response relationships, which has a significant impact on the way risk assessments are conducted for these chemicals (Dietrich et al. 2013; Bergman et al. 2013).

However, at the Berlin workshop, the protagonists of the scientific controversy were able to agree that the requirement for scientific criteria for the identification of endocrine disruptors per se, can be interpreted as an issue of hazard identification. This enabled the workshop participants to conclude that differences in opinion regarding the existence of thresholds and non-monotonic dose–response curves, although relevant to the risk characterization of EDs, are not a hindrance for defining scientific criteria for their identification. The consensus that was reached on scientific principles for the identification of endocrine-disrupting chemicals is offered as advice to the European Commission for the first step in their decision-making process to meet their legal obligations.

## Consensus statement

### Background

1. Key pieces of EU chemicals regulation, including the Plant Protection Product Regulation (EU No 1107/2009), the Biocidal Product Regulation (EU No 528/2012), the Water Framework Directive (2000/60/EC), REACH (EU No 1907/2006) and the Cosmetics Regulation (2009/1223/EC) include the aim of protecting human health and the environment from exposures to endocrine disruptors.
2. The European Commission (EC) is engaged in a process of elaborating specific scientific criteria for the identification of endocrine disruptors applicable to plant protection products and biocidal products. These criteria may have an impact on other pieces of EU legislation dealing with chemicals.
3. There are past and ongoing differences among scientists about the endocrinological, pharmacological and toxicological principles that should underpin scientific criteria for the identification of endocrine disruptors.
4. This paper represents an effort to establish a consensus among scientists who have taken part in these discussions. The initiative for this attempt came from a small group of scientists actively engaged in endocrine disruptor research. We map out an agreement about scientific principles that can underpin the identification of endocrine disruptors in the European Union (EU) according to the principle “One Substance—One Toxicological Assessment”.
5. The absence of accepted criteria for the identification of endocrine disruptors presents a significant stumbling block for a scientifically based regulation of endocrine disruptors that is enshrined in key pieces of EU chemicals regulation.
6. We acknowledge that there is a need in the EU to ensure continuation and enhancement of policies for the protection of human health and the environment from the effects of endocrine disruptors and those scientifically based criteria for the identification of endocrine disruptors are necessary for regulatory decision-making processes.
7. We recognize that the European Parliament, in its resolution of 14 March 2013 on the protection of public health from endocrine disruptors (P7_TA (2013)0091), took the view that these criteria should conform to the WHO/IPCS definition of endocrine disruptors, and should be based on the best available science.
8. The field of “endocrine disruptor research” draws from many scientific disciplines including physical chemistry, biochemistry, molecular biology, toxicology, pharmacology, endocrinology, developmental biology, epidemiology, clinical medicine and many others. Each of these fields can have a different language and a different logic to understand the unique complexities of their particular level of investigation.
9. We recognize that the views of scientists working at different levels of investigation and with different training and research experience may contribute to the appearance of a debate when the topic cuts across multiple disciplines. Therefore, we believe the most important consensus that we can achieve is one that relates to the principles, which should form the basis of the development of scientific criteria for the identification of endocrine disruptors [as requested by EU legislation].

### Definition of endocrine disruptors and related concepts

10. We acknowledge the WHO definition of an endocrine disruptor as follows: “An ED is an exogenous substance or mixture that alters the function(s) of the endocrine system and consequently causes adverse effects in an intact organism, or its progeny, or (sub) populations”.
  - Alterations of the function of the endocrine system may arise from interaction with hormone receptors, changes in circulating levels of the hormone, and from the impact of chemical(s) on hormone synthesis, transport, metabolism and other factors.
  - In the WHO definition, the term “adverse effect” refers to “A change in morphology, physiology, growth, reproduction, development or lifespan of an organism which results in impairment of functional capacity or impairment of capacity to compensate for additional stress or increased susceptibility to the harmful effects of other environmental influences”.
  - The term “intact organism” is understood to mean that the effect would occur in vivo, either observable in a test animal system, epidemiologically or clinically. However, it does not necessarily mean that the adverse effect has to be demonstrated in an intact test animal, but may be shown in adequately validated alternative test systems predictive of adverse effects in humans and/or wildlife. The importance of mechanistic data derived from experimental systems (in vitro or in vivo in which the animals have been surgically or genetically altered as part of a focused experiment) was also recognized.
11. We acknowledge that certain hormones interact with their receptors according to an equilibrium reaction. Accordingly, the concentrations of both free hormone and free receptor are important variables controlling hormone action, explaining why different cells and tissues at different times during development are differentially sensitive to the hormone.
12. Experimental work has led to a better understanding of the role of hormones in development and during the maintenance of physiological functions. We recognize that disruption of the programming role of hormones during prenatal and postnatal development can cause adverse effects that do not become evident until later in life.
13. Interference with the role of many hormones during the maintenance of physiological functions in adult life can also lead to adverse effects.
14. We resolve that the scientific knowledge about the principles that govern the induction of adverse effects by disrupting the programming function of hormones during development is sufficiently advanced to warrant regulatory action.

### Sources of uncertainty

15. We recognize that the identification of chemicals that contribute to adverse effects on human health is fraught with difficulties which, in the case of endocrine disruptors, can be traced to several specific factors. Many of the critical events discussed in the context of endocrine disruption in humans may occur in fetal life, during childhood or puberty. Exposures during these periods are often difficult to reconstruct, thus obscuring any causal relationships that may exist. Some chemicals whose health effects have been relatively well studied in other areas, have also been subjected to the assessment of endocrine effects, but other chemicals in widespread commercial use have not been evaluated so.
16. On the other hand, the existing framework of internationally validated test systems for the identification of endocrine disruptors must be further developed to ensure detection of health effects relevant to endocrine disruption in humans. For example, test systems suitable for the identification of effects consequent to many specific modes of action in disrupting the function of hormone systems are missing, although efforts to address these gaps are ongoing.
17. Non-monotonic dose–response relationships and low-dose effects of endocrine disruptors have been described in the literature. The implications of these observations for testing strategies and risk assessment continue to be debated, and we acknowledge the importance of this scientific discourse. However, we believe that a consensus about these issues is unlikely to emerge in the near future. Nevertheless, in our view the establishment of criteria for the identification of endocrine disruptors is possible without resolution of these issues.
18. We emphasize that these sources of uncertainty should not delay current efforts to regulate endocrine disruptors. Nevertheless, elucidation of the above issues which are significant sources of uncertainty will require considerable research efforts in the future. These efforts will be essential for scientifically based regulations of endocrine disruptors in key pieces of EU chemicals regulation.

### Scientific foundations of regulatory decision-making

19. The various relevant pieces of EU chemicals regulation require both hazard and risk assessment approaches^1^ to enable decision-making to be applied in different ways.
20. The identification of a compound as an endocrine disruptor is a hazard identification procedure. Established principles governing disruption of the programming function of hormones mean that hazard identification for endocrine disruption has to take account of the timing of exposure relative to life stage and that transient indices or effects should not necessarily be considered adverse.
21. We recognize that certain adverse outcomes appearing to arise from endocrine disruption can also occur through non-endocrine modes of action. Moreover, adverse effects or modes of action consistent with endocrine-disrupting characteristics but demonstrated to be non-specific effects secondary to another toxic effect are not considered appropriate for identification of endocrine disruption. The identification of a chemical as an endocrine disruptor therefore has to rely on weight-of-evidence evaluations of both adversity and mode of action together. We agree that endocrine activity on its own should not trigger a chemical’s identification as an endocrine disruptor.
22. We agree that a chemical’s potency to induce an adverse effect is an important factor for consideration during the characterization of the hazards of endocrine disruptors. However, potency is not relevant for identification of a compound as an endocrine disruptor. However, there may be high doses (e.g. the oral toxicity limit of 1000 mg/kg body weight/day) above which identification as an ED would not be warranted.
23. Criteria for identifying chemicals as endocrine disruptors would need to be accompanied by the implementation of relevant test systems in EU regulations. We note that many relevant OECD guidelines exist which have not yet been consistently integrated into the regulatory frameworks. There is lack of validated tests for a number of modes of actions. We recommend that respective EU directives, regulations and other relevant guidance are updated to incorporate validated and internationally agreed test systems for endocrine disruptors. In this context, guidance and scientific advice need to be updated to indicate how the outcome of those tests should be evaluated in the regulatory context, and to include endocrine pathways and adverse health effects that are insufficiently explored by current toxicological testing.
24. This document has focused on the identification of endocrine disruptors. However, the assessment of the corresponding risks on human health and wildlife would further require consideration of dose–response relationships, including potency, exposure assessment and risk characterization, including susceptible subpopulations, severity and reversibility of effects. This emphasizes the importance of the “One Substance—One Toxicological Assessment” philosophy, and has implications for data generation of both regulated and unregulated chemicals.

### Research needs

25. More effective regulation could be achieved by closing certain knowledge gaps. We recommend that these knowledge gaps should be identified through a systematic gap analysis. Notwithstanding such an analysis, we recognize that future research needs to include the following main areas:
  - Exposure assessment of EDs,
  - Epidemiological studies of EDs with accurate characterization of exposures during relevant time periods,
  - Experimental research to clarify ED modes- and mechanisms-of-action, to produce an improved understanding of the molecular events underlying adverse outcomes and to better understand whether irreversible effects of developmental programming are induced in a threshold-dependent manner or not, and
  - Test method and biomarker development, including validation, to ensure more sensitive and robust identification of EDs.
26. We recognize that exposure assessments based on effect measurements and/or on a wide range of chemicals have the potential to identify previously unrecognized endocrine disruptors.
27. Resolution of the issues of non-monotonic dose–response relationships and whether effects are threshold-dependent requires systematic efforts to understand the mechanisms underlying adverse effects of endocrine disruptors.
28. The existence of dose-thresholds for endocrine disruptors continues to be debated. We recognize that it may be difficult to distinguish a true threshold from an apparent threshold which merely arises from the limits of detection of the experimental system. Thus, the question of the existence of dose-thresholds for endocrine disruptors cannot be resolved through empirical dose–response studies alone, but has to rely on mechanistic investigations and increased knowledge on the functions and programming of the endocrine system during specific windows of sensitivity. Such research is not considered a prerequisite for the identification of endocrine disruptors, but it is necessary for their risk assessment.
29. Many assays, models and tools for the study of ED-related modes and mechanisms of action already exist, but have not been taken forward into the assay validation process. A systematic analysis is needed to establish which existing assays are ready for validation.
30. While many existing “scientific tools” could be refined into validated assays, suitable model systems and assays are missing for certain mechanistic aspects of endocrine disruption. Concerted research and development efforts are needed to fill these gaps and are being developed.
31. The Commission requires that animal testing should be reduced and avoided where possible. Hence, there is a need to develop approaches that can reliably detect endocrine-disrupting chemicals using non-animal methods, with at least the same reliability as current methods. Criteria will be necessary to determine the acceptability of such methods.

## Footnotes

1 The WHO IPCS definitions for the four steps in risk assessment: hazard identification, hazard characterization, exposure assessment and risk characterization, have been used throughout this document.

## Glossary

Chemicals: Natural and anthropogenic substances as defined by their chemical structures.
Chemicals legislation: Substance and product-based legislation aiming at minimizing environmental and health risks currently relevant to endocrine disruption such as plant protection products (EC 1107/2009), biocides (EU 528/2012), food additives (EC 1333/2008), REACH (EC 1907/2006), food contact materials (EU 10/2011), cosmetics (EC 1223/2009).
Endocrine disruptor: WHO definition: An endocrine disruptor is an exogenous substance or mixture that alters function(s) of the endocrine system and consequently causes adverse health effects in an intact organism, or its progeny, or (sub)populations.
Hazard identification: IPCS definition: The identification of the type and nature of adverse effects that an agent has an inherent capacity to cause in an organism, system or (sub)-population. Hazard identification is the first stage in hazard assessment and the first step in the process of risk assessment.
Hazard characterization: IPCS definition: The qualitative and, wherever possible, quantitative description of the inherent properties of an agent or situation having the potential to cause adverse effects. This should, where possible, include a dose–response assessment and its attendant uncertainties. Hazard characterization is the second stage in the process of hazard assessment and the second step in risk assessment.
One Substance—One Toxicological Assessment: A chemical that falls under several regulatory systems would have only one assessment, which would be accepted by all of the regulatory systems. This does not necessarily imply that the regulatory decision would be the same, which would depend on a number of considerations.
